# High-resolution computed tomography features of asbestosis versus fibrotic hypersensitivity pneumonitis: an observational study

**DOI:** 10.1186/s12890-022-01967-3

**Published:** 2022-05-25

**Authors:** Ruimin Ma, Shuang Li, Yuanying Wang, Shuqiao Yang, Na Bao, Qiao Ye

**Affiliations:** 1 Department of Occupational Medicine and Toxicology, Clinical Center for Interstitial Lung Diseases, Beijing Institute of Respiratory Medicine, Beijing Chao-Yang Hospital, Capital Medical University, No. 8 Workers’ Stadium South Road, Chao-Yang District, Beijing, China; 2 Department of Respiratory Medicine, Beijing Shunyi Hospital, Beijing, China; 3 Department of Respiratory Medicine, Beijing Chao-Yang Hospital, Capital Medical University, Beijing, China; 4 Department of Radiology, Beijing Chao-Yang Hospital, Capital Medical University, Beijing, China

**Keywords:** Asbestosis, Hypersensitivity pneumonitis, High-resolution computed tomography, Fibrosis, Pleura

## Abstract

**Background:**

Asbestosis and fibrotic hypersensitivity pneumonitis (FHP) share the pathogenetic mechanisms induced bronchiolocentric fibrotic process secondary to inhalation exposure. Under the occupational and environmental mixed exposures, asbestosis and FHP are needed to make the differential diagnoses on high-resolution computed tomography (HRCT), especially in the countries still using asbestos. The study aimed to analyze the HRCT features of asbestosis versus FHP.

**Methods:**

The patients with asbestosis or with HP were sequentially recruited in this comparative study at Beijing Chaoyang Hospital between January 2006 and December 2016. Patients’ clinical data were obtained from a predesigned charts. The international classification of HRCT for occupational and environmental respiratory diseases was used to categorize chest imaging findings in patients. The calculation of test statistics was used to compare the imaging features of asbestosis and FHP.

**Results:**

341 patients with asbestosis and 158 patients with HP were sequentially recruited, among which 204 patients with asbestosis and 74 patients with FHP were eligible for data analysis. Patients with asbestosis were older and had a longer latent period until disease manifestation than those with FHP. Asbestosis was characterized by irregular and/or linear opacities, with lower lung preponderance, accompanied by ground-glass opacities and mosaic attenuation. Notably, 98.5% of patients with asbestosis showed benign pleural abnormalities, and 39.7% of these patients had diffuse pleural thickening with parenchymal bands and/or rounded atelectasis. Abnormalities of the mediastinal and diaphragmatic pleura were observed only in cases of asbestosis, and this finding showed high specificity for the diagnosis for asbestosis compared with that for FHP. Subpleural dots or diaphragmatic pleural abnormalities showed moderate sensitivity and high specificity for diagnosis of asbestosis compared with that for FHP. Interobserver reliability was good for evaluation of imaging findings including honeycombing, pleural calcification, lymphadenectasis, and lymph node calcification.

**Conclusions:**

HRCT-based imaging findings can distinguish between asbestosis and FHP to a certain extent, particularly with regard to subpleural dots and diaphragmatic pleural abnormalities that characterize the former.

**Supplementary Information:**

The online version contains supplementary material available at 10.1186/s12890-022-01967-3.

## Background

Asbestos is a natural crystalline silicate mineral that has various commercial and industrial uses, such as in fire prevention and insulation. Asbestos is widely used in industrial production as well as in routine life; chrysotile fibers are the most common form of asbestos that accounts for > 90% of asbestos products used worldwide. The International Agency for Research on Cancer has classified asbestos as a group 1 carcinogen [1]. Asbestos-related diseases (ARDs) show a long latent period (30–60 years) [2]. Owing to the long latent period, lack of accurate and complete history regarding asbestos exposure and lack of awareness among physicians often present a diagnostic and differential diagnosis challenge. Also, there are legal and compensatory issues in asbestosis thus differentiating it from other pulmonary diseases is essential.

According to The American Thoracic Society (ATS) guidelines, asbestosis is similar to other diffuse pulmonary diseases and therefore needs to be differentiated from other pneumoconiosis, idiopathic pulmonary fibrosis (IPF), hypersensitivity pneumonitis (HP), and sarcoidosis, among other such conditions [3]. Prima facie, asbestosis and IPF are often indistinguishable with regard to imaging findings. The fibrotic pattern observed in asbestosis is patchy in nature and mimics that of usual interstitial pneumonia (UIP) [4]. UIP typically presents as honeycombing with or without peripheral traction bronchiectasis or bronchiolectasis, predominantly in the subpleural and basal areas of the lungs [5]. Inhaled asbestos fibers/particles are phagocytosed by macrophages and are transported to the pleura via the lymphatic channels. The inhaled particles are also deposited in the respiratory bronchioles and alveoli. Long-term deposition lead to fibrosis of the distal bronchi and interstitium of the lungs [3]. HP is caused by repeated inhaled exposure to organic and low-molecular-weight compounds. Fibrotic HP (FHP) is histopathologically characterized by inflammation and fibrosis with a bronchiolocentric distribution, and pleural involvement is rare [6]. FHP usually represents an immune response of the body to antigen inhalation [7]. To an extent, asbestosis and FHP have a similar pathogenetic mechanism because of the exposure. An endoscopic lung biopsy definitively distinguishes between asbestosis and FHP; however, obtaining sufficient lung tissue samples is challenging, and the samples may not satisfactorily and accurately establish the histopathological diagnosis. And surgical lung biopsy is difficult to propose widely because of it is invasive and often patients are too sick to undergo surgery. In this study, we aimed to compare features of chest high-resolution computed tomography (HRCT) which is a non-invasive diagnostic tool between asbestosis and FHP.

## Methods

### Study design

This comparative study included two groups and conforms to the Strengthening the Reporting of Observational Studies in Epidemiology guidelines [8].

### Patient selection

We sequentially recruited the patients with asbestosis and the patients with HP, who were newly diagnosed at Beijing Chaoyang Hospital between January 2006 and December 2016. Asbestosis was diagnosed based on the International Labor Organization classification criteria after multidisciplinary discussions [3, 9]. Asbestosis who was having the clinical pathway in our medical center to make the diagnosis, include a history of asbestos exposure, the images of dynamic CT scans, and/ or PET-CT, bronchoscopy, percutaneous lung or pleural biopsy, etc. when it is necessary. FHP was diagnosed based on the diagnostic criteria of HP [7, 10]. The clinical pathway to make a diagnosis of HP includes a history of various organic antigens exposure, specific IgG testing, HRCT appearance, cell counts, and differential analysis of bronchoalveolar lavage, endoscopic biopsies, or surgical lung biopsy (SLB). A highly confident diagnosis of fibrotic HP in our study was enrolled for analysis. Inflammatory HP and FHP were classified based on the criteria and the pathway [10]. When making the differential diagnosis of different diseases, the specificity and sensibility of the criteria to diagnose the diseases were variable. A conventionally multidisciplinary diagnosis per week for respiratory diseases was adopted for the difficult patients. Patients with uncontrolled pneumonia, tuberculosis, autoimmune diseases, heart failure, severe liver and kidney dysfunction, malignant tumors, unavailability of HRCT data, and those with inflammatory HP and acute exacerbation of HP were excluded from the study. All patients completed a standardized questionnaire regarding their occupational and environmental history; all jobs throughout an individual’s working life were considered.

This study was approved by the Institutional Ethics Committee for Human Research, Beijing Chaoyang Hospital. Written informed consent was obtained from all participants involved in the research.

### High-resolution computed tomography

HRCT was performed using the following parameters: 0.625-mm sections, 1-s scan time, and a 10-mm interval in the apex-base scans with both lungs visualized in the field of view. A respiratory imaging expert and an occupational disease expert independently evaluated the HRCT imaging findings in patients with asbestosis and HP. The radiologists were certified by the National Health Commission of China for the diagnosis of occupational respiratory diseases using a national criterion that is in line with ILO classification guidelines. The characteristics and distribution of lesions and the HRCT scores were determined after discussion. The International Classification of HRCT for Occupational and Environmental Respiratory Diseases (ICOERD) criteria were used to describe the chest imaging findings in each lung, which was divided into three zones extending between the apex and base [11]. Following is the overall distribution for each side and zone of the thorax: upper arch of the aorta and the area superior to it, middle arch of the aorta extending inferiorly to the inferior pulmonary vein, lower inferior pulmonary vein and lower region including the diaphragm. The upper, middle and lower lung regions on each side were scored using a 4-point scale (0, 1, 2, and 3), and the total score was calculated as the sum of the 6 lung regions. The scores range between 0 and 18. Lesions evaluated included rounded opacities, irregular and/or linear opacities, inhomogeneous attenuation, honeycombing, emphysema, large opacities, pleural abnormalities, subpleural dots, coarse honeycombing, and a three-density pattern. Based on the 2013 ATS/European Respiratory Society guidelines for the diagnosis of idiopathic interstitial pneumonias [12], chest HRCT patterns were classified into UIP, nonspecific interstitial pneumonia (NSIP), organizing pneumonia (OP), and unclassifiable interstitial pneumonia (unclassifiable IP).

### Pulmonary function test

All patients underwent pulmonary function tests based on the guideline of spirometry [13]. The following respiratory parameters were measured: forced vital capacity (FVC), forced expiratory volume in 1 s (FEV1), FEV1/FVC ratio, peak expiratory flow (PEF), maximum expiratory flow at 25% vital capacity (MEF25%), MEF50%, MEF75%, MEF25–75%, total lung capacity (TLC), residual volume (RV)/TLC ratio, and diffusing capacity of the lung for carbon monoxide using the single-breath method (DLCO SB).

### Statistical analysis

All statistical analyses were performed using the SPSS Statistics software, V.25 (IBM Inc, Chicago, Illinois, USA). The median with interquartile range was used for descriptive analysis, mean ± standard deviation was used for continuous variables, and counts with percentages were used for categorical variables. The t-test, Mann–Whitney U test, Chi-squared test, and Fisher’s exact test were used for intergroup comparison. The calculation of test statistics were used to determine the predictive value of HRCT imaging findings to distinguish between asbestosis and FHP. The confidence interval of likelihood ratios was calculated using the Simmel method [14]. The kappa coefficient (κ) was used to evaluate interobserver reliability of imaging findings, which was defined as follows: poor (0.00 < κ ≤ 0.20), fair (0.20 < κ ≤ 0.40), moderate (0.40 < κ ≤ 0.60), good (0.60 < κ ≤ 0.80), and excellent (0.80 < κ ≤ 1.00) [15]. All comparisons were two-sided, and *P* value < 0.05 was considered statistically significant.

## Results

### Patients’ demographic characteristics

**Fig. 1:**
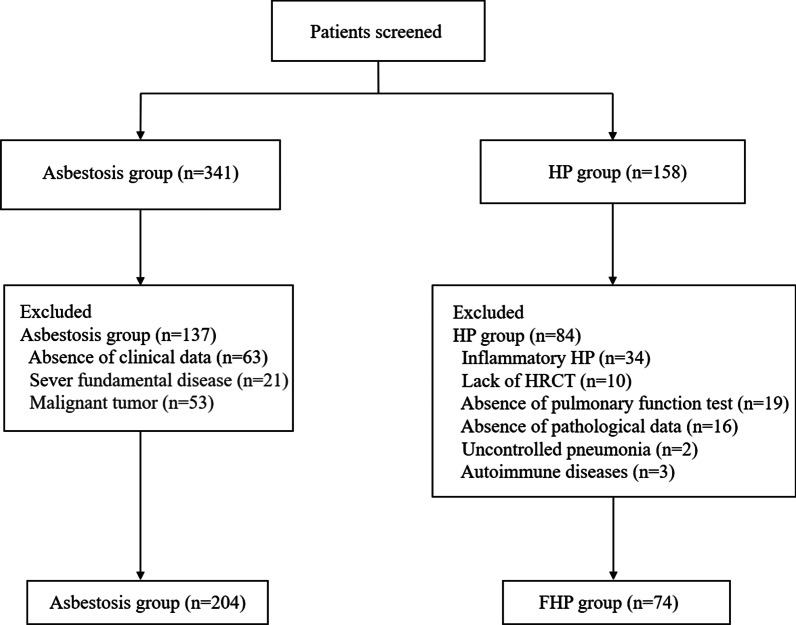
Flow chart of the enrolled population. We finally recruited 204 patients with asbestosis and 74 patients with FHP

341 patients with asbestosis were enrolled sequentially, of which 152 (44.6%) patients underwent BAL analysis, 204 patients with asbestosis were eligible for analysis (Fig. 1). Among 158 patients of HP screened, 142 (89.9%) patients had BAL cytology and lung histologic findings, and 74 patients with FHP were eligible for analysis shown in Fig. 1. Additional file 1: Table S1 showed demographic data of the study population. The age at diagnosis was younger, and the exposure time and latent period were shorter in patients with FHP than in patients with asbestosis (*P* < 0.001). No statistically significant intergroup differences were found in smoking habits. Of the 204 patients with asbestosis, 125 (61.3%) were employed in occupations associated with asbestos products, and 79 (38.7%) patients processed asbestos at home.

Occupations of patients with asbestosis included asbestos transport (2 [1.0%]) and asbestos processing and production (165 [80.9%]); of these, 124 (60.8%) patients were employed as asbestos weavers, 41 (20.1%) were involved in asbestos manufacture, and 37 (18.1%) were users of asbestos products, including operators, heat insulation workers, boiler maintenance workers, and mixers, among other such occupations. All patients with asbestosis had exposure to chrysotile fibers. Of the 74 patients with FHP, 55 (74.3%) had antigen exposure; 30 (40.5%) were animal antigens, of which 22 (29.7%) were associated with birds, and 8 (10.8%) were associated with pets such as cats and dogs, 19 (25.7%) were associated with microbial exposure, of which 9 (12.2%) were associated with humidifiers, hot baths, or humid environment, 9 (12.2%) were associated with crop farming processing or mushroom cultivation, and 1 (1.4%) was associated with sawdust exposure, and 6 (8.1%) were associated with hair dye use or isocyanate exposure. The antigen was unknown in 19 (25.7%) patients.

### Pulmonary function test results

Lung volume parameters including FVC% predicted (pred.) (*P* < 0.001), FEV1% pred. (*P* < 0.001), and small airway velocity indices including PEF% pred. (*P* < 0.001), MEF75% pred. (*P* = 0.001), MEF50% pred. (*P* < 0.001), and MEF25% pred. (*P* < 0.001) were lower in patients with asbestosis than in patients with FHP; however, no statistically significant intergroup difference was observed in TLC% pred., RV% pred., DLCO SB% pred., partial pressure of oxygen, and the composite physiologic index (*P* > 0.05 in all cases) (Table 1).

**Table 1:** Comparisons of lung function values between asbestosis and FHP patients

Characteristics	Asbestosis	FHP	*P* value*
n	204	74	
FVC (% predicted)	74.36 ± 20.44	85.99 ± 21.80	< 0.001
FEV1 (% predicted)	71.50 ± 21.19	83.30 ± 20.62	< 0.001
FEV1/FVC (%)	77.87 ± 9.75	80.49 ± 7.29	0.034
PEF (% predicted)	80.25 ± 25.88	99.97 ± 24.35	< 0.001
MEF75 (% predicted)	70.48 ± 33.26	86.23 ± 36.73	0.001
MEF50 (% predicted)	57.30 ± 27.31	72.60 ± 29.19	< 0.001
MEF25 (% predicted)	50.83 ± 26.34	64.90 ± 33.92	0.001
RV (% predicted)	91.18 ± 35.65	83.51 ± 27.11	0.136
TLC (% predicted)	78.80 ± 19.70	80.06 ± 18.17	0.629
RV/TLC (%)	47.91 ± 13.15	38.51 ± 9.78	< 0.001
DLCO SB (% predicted)	62.40 (39, 77.45)	53.20 (39.65, 69.25)	0.147
PaO_2_, mmHg (room air, at rest)	81.93 ± 14.16	78.97 ± 16.47	0.153
CPI	37.16 (25.39, 37.16)	40.13 (28.47, 51.98)	0.864

Data was presented as mean ± SD or median (IQR

*PFT* pulmonary function test, *FVC* forced vital capacity, *FEV1* forced expiratory volume in 1 s, *PEF* peak expiratory flow, *MEF25* maximum expiratory flow in 25% vital capacity, *MEF50* maximum expiratory flow in 50% vital capacity, *MEF75* maximum expiratory flow in 75% vital capacity, *RV* residual volume, *TLC* total lung capacity, *DLCO* diffusing capacity of the lung for carbon monoxide, *PaO*_*2*_ partial pressure of oxygen, *CPI* composite physiologic index, *FHP* fibrotic hypersensitivity pneumonitis

^*^P value: Asbestosis versus FHP

### Comparisons of high-resolution computed tomography findings between asbestosis and fibrotic hypersensitivity pneumonitis

The scores of irregular and/or linear opacities were lower in the asbestosis than in the FHP group (4.0 [2.0–8.0] vs. 8.5 [6.0–12.0], *P* < 0.001). The prevalence of subpleural lines < 5 mm from the pleura (26.0% vs. 6.8%, *P* < 0.001) and subpleural dots (56.9% vs. 13.5%, *P* < 0.001) was higher in the asbestosis than in the FHP group (Table 2). With regard to the distribution of irregular and/or linear opacities, the lower lung area was more commonly involved in the asbestosis group (Additional file 1: Fig. S1), whereas the middle and upper areas were more commonly involved in the FHP group. The percentage of peripheral involvement was higher in the asbestosis than in the FHP group (76.0% vs. 35.1%, *P* < 0.001). Basal honeycombing was common in the asbestosis and upper lung honeycombing was common in the FHP group. Inhomogeneous attenuation (ground-glass opacities) was more common in the FHP group. We observed no significant intergroup difference in the percentage of mosaic perfusion and three-density pattern (*P* > 0.05 in all cases) (Additional file 1: Fig. S2). With regard to pleural abnormalities, parenchymal bands, rounded atelectasis (Additional file 1: Fig. S3), and visceral, mediastinal, and diaphragmatic pleural abnormalities were observed only in the asbestosis group (Table 3). Prevalence of NSIP and OP was lower (*P* = 0.047, *P* < 0.001 respectively) and unclassifiable IP was higher in the asbestosis than in the FHP group (*P* = 0.002). No significant intergroup difference was found in UIP (*P* > 0.05) (Table 4).

**Table 2:** Pulmonary interstitial and parenchyma features between asbestosis and FHP on HRCT

Characteristics	Asbestosis	FHP	*P* value*
n	204	74	–
Rounded opacities	10 (4.9)	0	0.067
Irregular and/or linear opacities	200 (98.0)	74 (100)	0.576
Interlobular opacities	192 (94.1)	74 (100)	0.040
Intralobular opacities	159 (77.9)	25 (33.8)	< 0.001
Subpleural lines	56 (27.5)	9 (12.2)	0.008
Subpleural dots	116 (56.9)	10(13.5)	< 0.001
Honeycombing	19 (9.3)	6 (8.1)	0.756
Inhomogeneous attenuation	131(64.2)	69 (93.2)	< 0.001
Ground glass opacity	123 (60.3)	69 (93.2)	< 0.001
Mosaic attenuation	45(22.1)	15 (20.3)	0.749
Three-density pattern	31 (15.2)	13 (17.6)	0.632
Emphysema	43 (21.1)	18 (24.3)	0.563
Traction bronchiectasis	48 (23.5)	24 (32.4)	0.134

Data was presented as n (%)

*FHP* fibrotic hypersensitivity pneumonitis, *HRCT* high-resolution computed tomography

^*^P value: Asbestosis versus FHP

**Table 3:** Pleural abnormality between asbestosis and FHP on HRCT

Characteristics	Asbestosis	FHP	*P* value*
n	204	74	–
Pleural abnormality	201 (98.5)	57 (77.0)	< 0.001
Parietal type	120 (58.8)	57 (77.0)	0.005
Visceral type	81 (39.7)	0	< 0.001
Parenchymal bands	77 (37.7)	0	< 0.001
Rounded atelectasis	12 (5.9)	0	0.040
Distribution			
Chest wall	200 (98.0)	57 (77.0)	< 0.001
Mediastinum pleural	66 (32.4)	0	< 0.001
Diaphragm pleural	121 (59.3)	0	< 0.001
Pleural calcification	141 (69.1)	0	< 0.001
Chest wall	140 (68.6)	0	< 0.001
Mediastinum pleural	53 (26.0)	0	< 0.001
Diaphragm pleural	100 (49.0)	0	< 0.001
Pleural effusion	19 (9.3)	0	< 0.001

Data was presented as n (%)

*FHP* fibrotic hypersensitivity pneumonitis, *HRCT* high-resolution computed tomography

^*^P value: Asbestosis versus FHP

**Table 4:** Classification of the IIPs according to 2013 American Thoracic Society/European

HRCT pattern	Asbestosis	FHP	*P* value*
n	204	74	–
UIP, n (%)	20 (9.8)	8 (10.8)	0.805
NSIP, n (%)	38 (18.6)	22 (29.7)	0.047
OP, n (%)	0 (0)	6 (8.1)	< 0.001
Unclassifiable IP, n (%)	146 (71.6)	38 (51.4)	0.002

Data was presented as n (%)

*IIPs* idiopathic interstitial pneumonias, *HRCT* high-resolution computed tomography, *FHP* fibrotic hypersensitivity pneumonitis, *UIP* usual interstitial pneumonia, *NSIP* nonspecific interstitial pneumonia, *OP* organizing pneumonia

*P value: Asbestosis versus FHP

### Comparisons of the predictive value of high-resolution computed tomography findings between the asbestosis and fibrotic hypersensitivity pneumonitis groups

HRCT showed high sensitivity (0.94) and low specificity (0) for detection of interlobular opacities and high specificity (0.88, 0.86, and 0.92) but low or moderate sensitivity (0.27, 0.57, and 0.09) for detection of subpleural lines, subpleural dots, and honeycombing, respectively in asbestosis (Table 5). HRCT showed high specificity (0.80 and 0.82, respectively) but low sensitivity (0.22 and 0.15, respectively) for detection of mosaic attenuation and three-density pattern in asbestosis. Visceral, mediastinal, and diaphragmatic pleural involvement and pleural effusion are specific signs associated with asbestosis. Detection of subpleural dots and diaphragmatic pleural abnormalities showed high predictive value for diagnosis of asbestosis versus FHP (Tables 5 and 6).

**Table 5:** Identifying asbestosis and FHP in pulmonary interstitial and parenchyma features on HRCT

Characteristics	Sensitivity	Specificity	PPV	NPV	+ LR (95% CI)	− LR (95% CI)
Irregular and/or linear opacities						
Interlobular opacities	0.94	0	0.72	0	0.94 (0.91–0.97)	–
Subpleural lines	0.27	0.88	0.86	0.31	2.26 (1.18–4.33)	0.83 (0.73–0.93)
Subpleural dots	0.57	0.86	0.92	0.42	4.21 (2.34–7.58)	0.50 (0.42–0.60)
Honeycombing	0.09	0.92	0.76	0.27	1.15 (0.48–2.77)	0.99 (0.91–1.07)
Inhomogeneous attenuation						
Mosaic attenuation	0.22	0.80	0.75	0.27	1.10 (0.65–1.83)	0.98 (0.85–1.12)
Three-density pattern	0.15	0.82	0.70	0.26	0.87 (0.48–1.56)	1.03 (0.91–1.16)
Ground glass opacity	0.60	0.07	0.64	0.06	0.65 (0.57–0.73)	5.88 (2.48–13.93)

The PPV represents the patients who were diagnosed as asbestosis

*HRCT* high-resolution computed tomography, *PPV* positive predictive value, *NPV* negative predictive value, + *LR* positive likelihood ratio, *− LR* negative likelihood ratio, *CI* confidence interval

**Table 6:** Identifying asbestosis and FHP in pleural abnormalities on HRCT

Characteristics	Sensitivity	Specificity	PPV	NPV	+ LR (95% CI)	− LR (95% CI)
Pleural abnormality	0.98	0.23	0.78	0.85	1.28 (1.13–1.45)	0.06 (0.02–0.21)
Parietal type	0.59	0.23	0.68	0.17	0.76 (0.65–0.91)	1.79 (1.15–2.81)
Visceral type	0.40	1.00	1.00	0.38	–	0.60 (0.54–0.67)
Distribution						
Chest wall	0.98	0.23	0.78	0.81	1.27 (1.12–1.44)	0.09 (0.03–0.25)
Mediastinum pleural	0.32	1.00	1.00	0.35	–	0.68 (0.62–0.74)
Diaphragm pleural	0.59	1.00	1.00	0.47	–	0.41 (0.35–0.48)
Pleural effusion	0.09	1.00	1.00	0.29	–	0.91 (0.87–0.95)

The PPV represents the patients who were diagnosed as asbestosis

*HRCT* high-resolution computed tomography, *PPV* positive predictive value, *NPV* negative predictive value, + *LR* positive likelihood ratio, *−* *LR* negative likelihood ratio, *CI* confidence interval

### Accuracy of high-resolution computed tomography based diagnosis

HRCT images tended to show inconsistencies in the evaluation of rounded opacities, irregular and/or linear opacities, intralobular opacities, subpleural lines, subpleural dots, ground-glass opacities, and centrilobular emphysema. HRCT showed moderate diagnostic accuracy for rounded atelectasis, mediastinal and diaphragmatic pleural involvement, calcification of the mediastinal pleura, and pleural effusion. Notably, HRCT showed good diagnostic accuracy for honeycombing, pleural calcification, calcification of pleura on the chest wall and diaphragmatic pleura, as well as lymphadenopathy and calcification. Other signs were poor. The interobserver reliability was good for classification of UIP, NSIP, and unclassifiable IP based on chest HRCT imaging patterns.

## Discussion

This retrospective comparative study included 204 patients with asbestosis and 74 patients with FHP who underwent chest HRCT and pulmonary function tests. Patients with asbestosis were older and had a longer latent period than those with FHP. Asbestosis was characterized by irregular and/or linear opacities, with basal preponderance, accompanied by ground-glass opacities, and mosaic attenuation. Pleural abnormalities were observed in 98.5% of patients with asbestosis and of these, 39.7% of patients had diffuse pleural thickening with parenchymal bands and rounded atelectasis. Mediastinal and diaphragmatic pleural involvement occurred only in asbestosis, and HRCT showed high specificity for the detection of these pleural abnormalities for the diagnosis of asbestosis. HRCT showed moderate sensitivity and high specificity for detection of subpleural dots and diaphragmatic pleural abnormalities to distinguish between asbestosis and FHP.

Pleural plaque formation represents the most common pathological lesion in asbestos-induced pleural abnormalities [3]. Pleural inflammation, collagen deposition, and calcification may occur following exposure, and these changes manifest as pleural plaques even at low levels of asbestos exposure [3]. Fibrotic bands and peribronchiolar and alveolar fibrosis often coexist with pleural plaques; however, the association is not absolute [16]. We observed that in our study, 69.1% of the asbestosis showed pleural plaques and were not found in FHP. In this study, the percentage of asbestosis-induced pleural abnormalities was significantly higher than that of FHP-induced pleural lesions. Pleural abnormalities (particularly the visceral type) are a distinctive manifestation of asbestosis, which is also referred to as diffuse pleural thickening. Visceral pleural thickening includes parenchymal band formation and rounded atelectasis. Pleural thickening tends to occur bilaterally and is patchy, although it may be unilateral in 33% of patients [3]. Studies have shown that parenchymal bands and diffuse pleural thickening are often associated with visceral pleural fibrosis. Parenchymal bands are known to be of diagnostic value in asbestosis complicated by pleural disease [17]. Rounded atelectasis is caused by thickening of the visceral pleura and collapse of the central lung parenchyma and is often associated with inflammatory pleural disease and may mimic a tumor on chest radiography. In our study, parenchymal bands and rounded atelectasis only occurred in asbestosis; these imagings may be the signs to diagnose the asbestosis combined with pleural disease.

FHP tends to primarily affect the middle and upper lungs and is characterized by decreased lobule density, decreased blood flow, and centrilobular nodules [18]. Asbestosis mainly occurs in the lower segments of the lungs. Akira et al. [19] reported that asbestosis affected the lower segments of the lungs in 78 (98%) of the 80 patients investigated in the study, and only 2 patients showed findings in the upper lungs. Lower lung involvement in asbestosis is attributable to the fact that asbestos fibers easily enter the terminal bronchioles. Owing to the effect of gravity, asbestos fibers are deposited in the lower lung, leading to the typical pattern of distribution observed in cases of asbestosis. These distributions were the same as the pathogenetic mechanism. Previous studies have shown that 94%, 85%, and 26% of patients with asbestosis presented with subpleural dots, subpleural lines, and mosaic perfusion, respectively [20]. Among the 204 patients with asbestosis secondary to chrysotile fiber exposure investigated in our study, 56.9%, 27.5%, and 22.1% of patients showed subpleural dots, subpleural lines, and mosaic attenuation on chest HRCT. Thus the most common seen in asbestosis was the subpleural dots. Subpleural dot and line formation may be associated with chrysotile fibers, which are more likely to get deposited at the distal end of the airways during respiration. Histopathologically, subpleural dots represent peribronchiolar nodular fibrosis involving the alveolar ducts [21]. Bronchiolar wall thickening and flattened and collapsed alveoli manifest as subpleural lines [22].

Uneven pulmonary perfusion due to airway or vascular disease is referred to as a mosaic attenuation pattern. Mosaic attenuation is an important CT-based imaging finding that aids in detection of IPF and diagnosis of FHP [23]. Asbestosis affects the small airways [24], and it is unclear whether mosaic attenuation can successfully distinguish between asbestosis and FHP. In this study, we observed no statistically significant difference in the percentage of mosaic attenuation between the asbestosis and FHP groups, and this can be shown clearer by the execution of expiratory phase CT acquisition. HRCT showed that inhomogeneous attenuation was observed in up to 64.2% of patients with asbestosis, in addition to ground-glass opacities and mosaic attenuation; specifically, 68.9% of patients with mosaic attenuation showed a “three-density pattern” sign. Radiologist Webb first described the “three-density pattern” sign, which refers to an imaging finding of low-density lobules, preserved lobules, and air trapping [23]. A survey-based study by Delphi emphasizes the significance of this sign for the diagnosis of FHP [25]. Asbestosis is histopathologically characterized by peribronchiolar and subpleural fibrosis. A few patients may present with UIP-type lesions, usually accompanied by benign pleural abnormalities, and asbestos bodies may be identified in the lung tissue [26]. In the present study, unclassifiable IP was commonly observed in cases of asbestosis. FHP represents a lung allergy caused by exposure to various antigens, and the imaging findings may manifest as UIP, NSIP, OP, bridging fibrosis, or central bronchiolar fibrosis with bronchiolar metaplasia.

Following are the limitations of this study: (a) The single-center retrospective study design (204 and 74 patients with asbestosis and FHP, respectively) may be associated with a selection bias. (b) The small sample size and the small number of patients with some imaging patterns may have affected the statistical results. (c) The patients’ samples between the two groups were unbalanced because the present study was just a comparative study, so in the future, a large-scale study is needed to carry out. (d) We compared only asbestosis and FHP in this study. Asbestosis also needs to be differentiated from other occupational interstitial lung diseases. (e) HRCT showed poor sensitivity and high specificity for detection of subpleural lines, honeycombing, mosaic attenuation, and a three-density pattern. Therefore, asbestosis and FHP still have the value of differential diagnosis. (f) The cumulative exposure is broadly represented by the duration of asbestos exposure, and field monitoring data are unavailable for patients with chrysotile exposure.

## Conclusions

This study highlights the similarities and differences in chest imaging findings between patients with asbestosis and FHP; we observed that pleural abnormalities, parenchymal bands, and rounded atelectasis showed high diagnostic value for asbestosis. Subpleural dots and diaphragmatic pleural abnormalities can distinguish between asbestosis and FHP to a certain extent. In addition to representing a serious occupational health concern in China, asbestos exposure causes environmental pollution and is a threat to human health. Owing to the long latent period, the health hazards associated with asbestos tend to persist even after being banned in several regions. It is necessary to improve the diagnostic accuracy of modalities, particularly of chest imaging findings, to facilitate early diagnosis and prompt initiation of comprehensive treatment. We also have some limitations in our study, so further large-scale study about the HRCT features between the asbestosis and FHP is essential.

## Supplementary Information

**Additional file 1.** Methods and file figures.

## Data Availability

The datasets used and/or analysed during the current study are available from the corresponding author on reasonable request.

## Abbreviations

FHP: Fibrotic hypersensitivity pneumonitis
HRCT: High-resolution computed tomography
κ: Kappa coefficient
IARC: International agency for research on cancer
ARDs: Asbestos related diseases
IPF: Idiopathic pulmonary fibrosis
UIP: Usual interstitial pneumonia
STROBE: Strengthening the reporting of observational studies in epidemiology
ILO: International Labor Organization
ILDs: Interstitial lung diseases
CTD: Connective tissue disease
ICOERD: International classification of HRCT for occupational and environmental respiratory diseases
ATS: American Thoracic Association
ERS: European Respiratory Society
IIPs: Idiopathic interstitial pneumonias
NSIP: Nonspecific interstitial pneumonia
OP: Organizing pneumonia
FVC: Forced vital capacity
FEV1: Forced expiratory volume in 1 s
PEF: Peak expiratory flow
MEF: Maximum expiratory flow
TLC: Total lung capacity
RV: Residual volume
DLCO: Diffusing capacity of the lung for carbon monoxide
PaO_2_: Partial pressure of oxygen
CPI: Composite physiologic index
SD: Standard deviation
IQR: Inter quartile range
PPV: Positive predictive value
NPV: Negative predictive value
+LR: Positive likelihood ratio
−LR: Negative likelihood ratio
CI: Confidence interval
