# Supplementary material for: High-resolution computed tomography features of asbestosis versus fibrotic hypersensitivity pneumonitis: an observational study
Source: BMC Pulm Med. 2022 May 25;22:207. doi: 10.1186/s12890-022-01967-3 (PMC9131664; doi:10.1186/s12890-022-01967-3)
Supplement: Supplementary file 1 — Additional file 1. Methods and file figures. [file 12890_2022_1967_MOESM1_ESM.docx]

**Additional files**

**Methods**

**Patient selection**

All work history, including any previous work, was collected by questionnaire for all patients. Our hospital is the largest occupational disease prevention center in our city, 20 kilometers away from the asbestos products factory opened from 1950s to 1970s. The duration of exposure (number of years) was determined due to the lack of atmospheric measurements and detailed information on the frequency of exposure to asbestos for each job.

**Additional file figures**


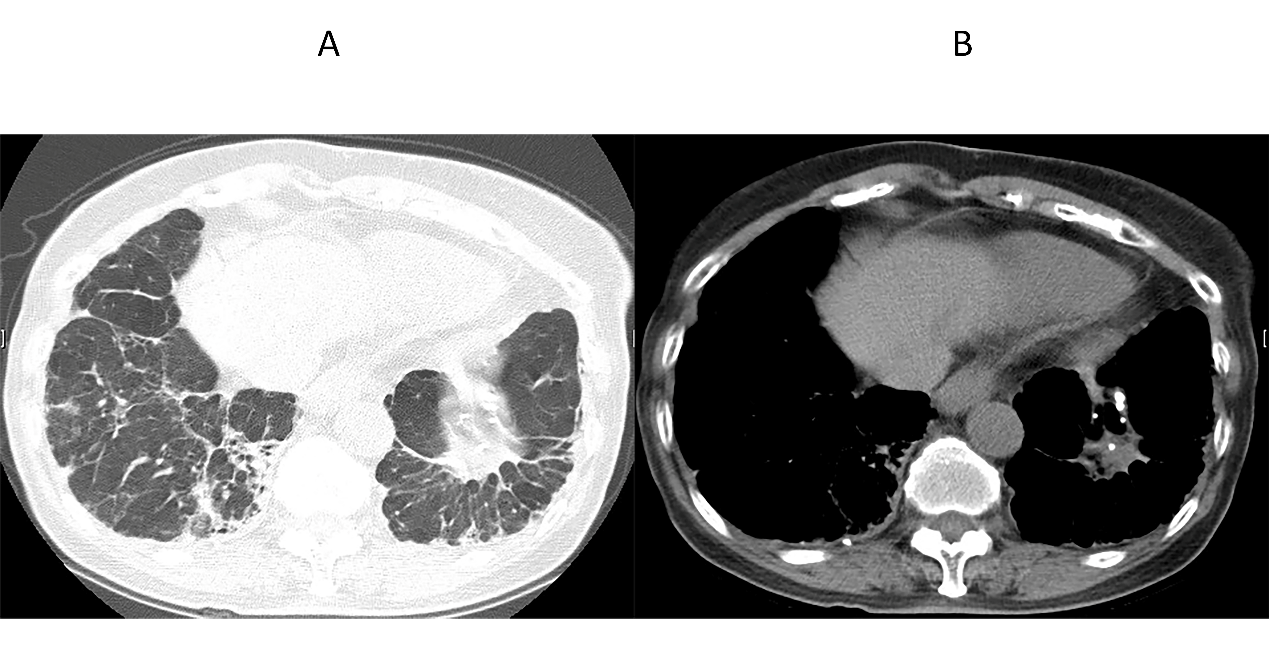


**Additional file 1: Figure S1** Representative case of asbestosis in a 66-year-old woman (weaver by occupation). Chest HRCT showing (A) interlobular septal thickening, ground-glass opacities, and mosaic attenuation, (B) pleural thickening with calcification.

HRCT: high-resolution computed tomography


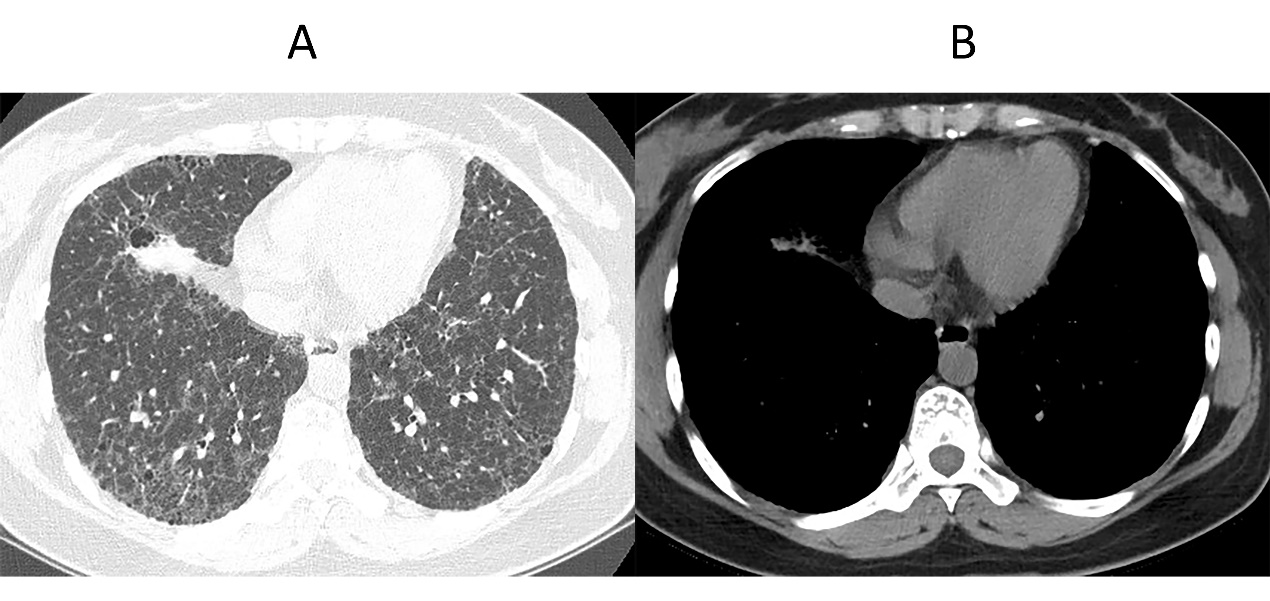


**Additional file 1: Figure S2** FHP in a 36-year-old woman, who grew corn at home and participated in the annual harvest. Chest HRCT showing (A) multiple thin-walled cystic cavities, interlobular septal thickening, intralobular interstitial thickening, ground-glass opacities, diffuse rounded opacities, with a centrilobular distribution, and “three-density pattern”, (B) the right plural is thickening.

FHP: fibrotic hypersensitivity pneumonitis, HRCT: high-resolution computed tomography


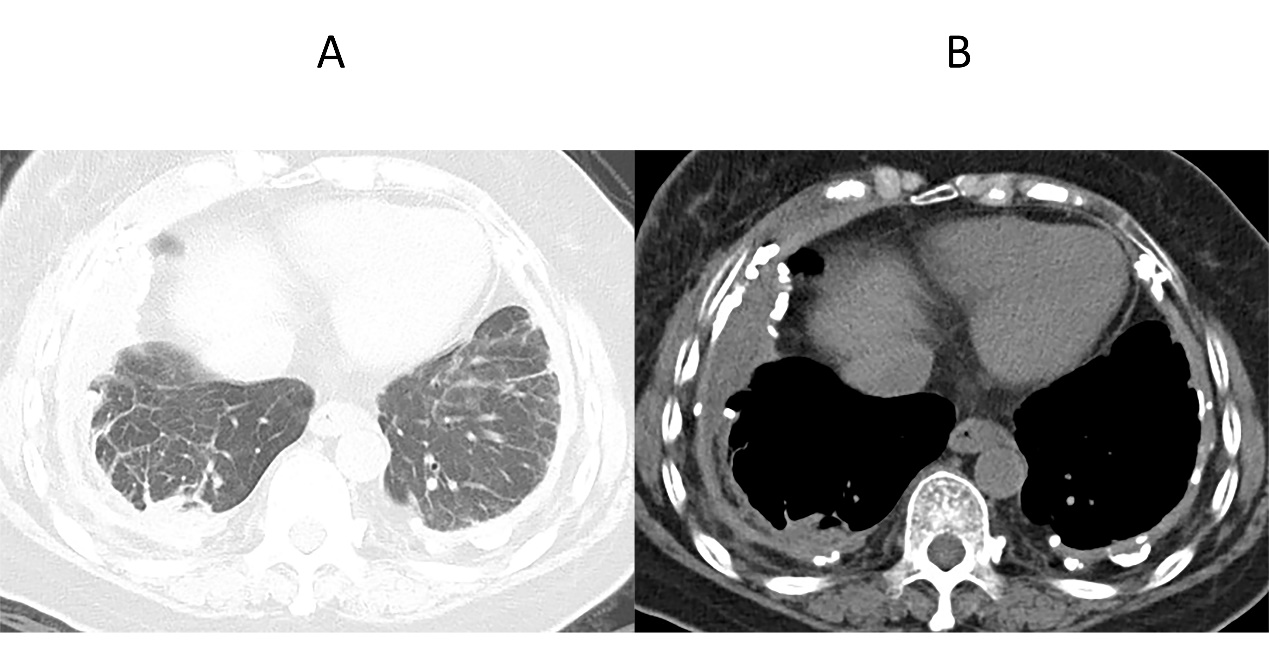


**Additional file 1: Figure S3** Asbestosis in a 64-year-old woman (weaver by occupation). Chest HRCT showing (A) interlobular septal thickening with rounded atelectasis and, (B) diffuse pleural thickening with calcification.

HRCT: high-resolution computed tomography

| **Additional file 1: Table S1** Demographics of the enrolled population | | | |
| --- | --- | --- | --- |
|  | Asbestosis | FHP | *P* value ^*^ |
| n | 204 | 74 |  |
| Age (years) | 68.9±9.0 | 55.4±11.7 | <0.001 |
| Female (n, %) | 122 (59.8) | 43 (58.1) | 0.903 |
| BMI (kg/m^2^) | 25.5±3.5 | 24.0±3.3 | 0.382 |
| Smoking status |  |  |  |
| Current smokers, (n, %) | 50 (24.5) | 21 (28.4) | 0.513 |
| Cumulative pack-years | 17.5(7.13,31.25) | 20(12.5,30) | 0.415 |
| Smokers≥20 pack-years, (n, %) | 25(12.3) | 13(16.3) | 0.445 |
| Duration of exposure (years) | 14 (6,22) | 10 (1,15) | <0.001 |
| Latent period (years) | 49 (40,54) | 10 (1.5,16) | <0.001 |

Date was presented as mean ± SD, median (IQR) or n (%).

Abbreviations: BMI, body mass index; FHP, fibrotic hypersensitivity pneumonitis.

^*^*P* value：Asbestosis vs FHP.
